# Is Dating Behavior in Digital Contexts Driven by Evolutionary Programs? A Selective Review

**DOI:** 10.3389/fpsyg.2022.678439

**Published:** 2022-02-28

**Authors:** Jorge Ponseti, Katharina Diehl, Aglaja Valentina Stirn

**Affiliations:** Institute of Sexual Medicine and Forensic Psychiatry, Medical School, Kiel University, Kiel, Germany

**Keywords:** sexual strategies, evolutionary psychology, dating platforms, dating apps, sexual selection, digital sexual services

## Abstract

In recent years, millions of citizens all over the world have used digital dating services. It remains unknown to what extent human sexuality will be changed by this. Based on an evolutionary psychological perspective, we assume that sexual selection shaped behavioural tendencies in men and women that are designed to increase the reproductive fitness. These tendencies are referred to as sexual strategies. Males and females sexual strategies differ according to sex-dimorphic reproductive investments. We assume that this inheritance will affect human sexuality also in a digital future. To evaluate this assumption, we conducted a selective review of studies on digital dating services. Based on sexual selection theory, we derived a number of hypotheses regarding how men and women will use digital dating services typically and how the use of digital dating services might affect sexual wellbeing. Out of an initial data set of 2,568 records, we finally reviewed a set of 13 studies. These studies provided support for the notion that men and women act in the digital dating area according to sex-typical strategies. However, sometimes the circumstances of digital dating affect communication flow, e.g., in that men are even more active in establishing contacts than they are in real world conditions. Overall, women appear to accomplish their sexual goals in digital dating arenas more than men do given a surplus of male demand. Our results suggest that future human sexuality will be impacted by an interaction of both: sex-dimorphic ancient sexual strategies and new technologies.

## Introduction

Digital applications have widely influenced everyday life in most human societies. Moreover, there is good reason to assume that this holds true for the sexual life histories of many citizens in modern societies as well. For example, dating platforms have millions of users. Thirty percent of German adults have used a digital dating service at some point (Statistica, 2020). About 41% of adults in Germany have used digital services to send erotic images of themselves (Döring and Mohseni, 2018). Twelve per cent of all Internet calls in Germany are searches for adult content (Arthur, 2013). In other countries, such as the United States, 46% of singles used online dating to find a new partner, and 1/3 of all couples who married between 2005 and 2012 in the United States met online (Jung et al., 2019).

It is possible that online sexual activities diverge from offline sexual behaviour. Dating platforms offer highly differentiated services addressing a growing variety of sexual contacts. People engage in long-distance sexual activities like cybersex via webcam, chatting with sex bots or interacting with sex robots. The division between porn users and porn producers has also vanished since platforms that broadcast users’ own sexual activities are provided. In light of these new developments, one might argue that digital sexual services will change human sexuality in the future. Some observations, however, call this assumption into question. In modern gender-equal societies, gender-stereotyped personality traits and gender-stereotyped job careers are more frequent in comparison to more traditional gender unequal societies (Stoet and Geary, 2018; Mac Giolla and Kajonius, 2019). These findings suggest that people actualise personality traits that are shaped by evolutionary selection when culture allows them to freely develop their personal aptitudes and personality. With respect to digital sexual services, we therefore expect that men and women use digital dating tools according to gender dimorphic sexual strategies.

In the following, first we will outline some basic ideas of sexual selection theory (Andersson, 2019), and second we will derive hypotheses of how ancient sexual strategies might affect the use of digital sexual services today.

Sexual selection, as one specific case of natural selection, operates on variance in reproductive success. If individuals differ in mating success, traits that aid their reproductive success will find their way to subsequent generations more frequently. Hence, adaptions will follow sexual selection. Traits that aid reproductive success differ between the sexes. Given that the more investing sex, i.e., females in mammals, needs more time for any reproductive act (because of gestation and lactation), sexually receptive females become scarce relative to males. Because of female scarcity, males more than females are selected for their ability of effectively compete for mates. In consequence, in males variability in reproductive success is higher than in females, not only in mammals (Brown et al., 2009; Boyd and Silk, 2020, p. 147) but also in insects (Bateman, 1948). Therefore, sexual selection operates more on males than on females. As sexual selection pressure operates differently between the sexes, the resulting adaptions – in anatomy and behaviour – are sex dimorphic. Traits that help males to increase the number of mates will be favoured by selection. Females in contrast can easily approach the maximum reproductive capacity of their sex (about 15 children in women and many thousands in men), which leads to a lower variability in the number of offspring within the female sex. However, there is more within-sex variability in how many offspring survive. Females cannot increase their reproductive fitness by increasing the number of mates but by increasing the quality of their mates (either in terms of their “genetic quality” and/or in terms of the resources males are able and willing to invest in their partner and offspring). Sexual selection theory predicts choosiness as a typical sexual strategy of females and strategies to increase the number of mates as a typical sexual strategy of males (i.e., stronger competition for mates, monopolisation of females, preference for young and sexually attractive females, interest in casual sex, short-term mating and sex with low investment). Males can increase their reproductive success not only by mating with numerous females but also by mating with females with a high reproductive capacity, namely young and attractive females.

Numerous studies across various cultures have provided empirical support for the assumption that sexual strategies of men and women follow the predictions of sexual selection theory. Women show a higher preference than men for mates who possess resources and are willing and interested in investing in children; men, in contrast, show a higher preference than women for physically attractive and young mates (Kenrick et al., 1994; Li et al., 2002; Brase, 2006; Roney et al., 2006; Buss and Shackelford, 2008; Conroy-Beam et al., 2015). Same sex competition usually occurs along those dimensions that are sexually preferred by the opposite sex. This allows individuals that own much of the sexually preferred cues to be more choosy and do gain more easily there sexual goals. Hi attractive women were found to be particularly choosy (Buss and Shackelford, 2008) and high status men look more for younger mates than lower status men do (Grammer, 1992). Given that a high number of sexual mates is associated with fitness gains in males more than in females, women are less prone to engage in short-term sex than men are (Clark and Hatfield, 1989; Schmitt, 2005; Voracek et al., 2005; Gueguen, 2011). If women engage in short-term sex, they are particularly attracted by cues of masculinity (i.e., cues for “good genes”) like tallness, physical strength and deep voice pitch (Puts, 2005; Roney et al., 2006). Women are more motivated to engage in short-term sex when there is a possibility to take fitness benefits out of these “good genes,” i.e., when they are in the ovulatory phase of the menstrual cycle (Baker and Bellis, 1995; Pillsworth and Haselton, 2006).

In natural environments, the operational sex ratio (i.e., the number of males relative to the number of females in a given mating area) influences the sexual strategies of males and females. If there is a surplus of females, sexual competence between women increases and women tend to lower the preconditions for sex. Women engage more in short-term sex when there is a surplus of women – sex becomes cheap. In the opposite case, women’s willingness to engage in short-term sex decreases (Barber, 2000; Schmitt, 2005). The impact of the operational sex ratio in how men and women pursue their sexual strategies illustrates the interaction between evolved behavioural tendencies and environmental circumstances. We assume that this interactive mechanism will also affect mating behaviour in digital sexual contexts. Given that there is a variety of digital dating services – some of them advertise rather casual sex, others advertise high quality mates and long-term commitment – possibilities to gain sexual goals for men and women will depend on these factors as well. However, in general we assume that the enhanced interest in many sexual mates in men will put women in digital mating markets in a more powerful position compared to men. In natural dating environments it was found that women send inconspicuously cues to men thereby controlling the “first step” of men’s courtship behaviour (Moore and Butler, 1989). In the absence of female non-verbal steering in online dating markets, we assume that a prevalence of male initiative will be more pronounced in online dating markets than in natural environments. This is also in accordance with notions of many of our students and patients: typically, men lament that their request were rarely responded while women complain about being overwhelmed by request, even without having a portrait of themselves in their account. If such a surplus of male demand is given, this will allow women to control the interaction with their male counterparts more according to their own needs. Therefore, we expect women to be more self-centred and self-oriented when communicating with men in online dating platforms. Furthermore, a surplus of male demand will also allow older women (that are less attractive to males in natural dating environments) to improve their dating chances.

Given the absence of some natural restraints in digital encounters (thereby allowing users to interact simultaneously and anonymously with multiple potential mates) we furthermore expect that using digital dating tools can lead to an amplification of sexual benefits as well as harm. Possible benefits might be an increased probability of finding a good mate in consequence of having access to a wider mating pool. Psychological harm can be the result of sexual conflicts. In evolutionary psychology sexual conflicts refer to those instances in which one individual tries to realise its sexual strategies (i.e., maximise its fitness) at the cost of its partners sexual strategies (respectively the partners fitness) (Buss, 1989a). There are numerous examples of how sexual strategies can interfere when men and women interact: ranging from deception about emotional commitment, deception about sexual fidelity, deception about willingness or ability to provide resources, deception about attractiveness, deception about fatherhood, or strategies to circumvent female choosiness by use of coercion. In natural environments, men and women have evolved contra strategies to protect against the harmful consequences of sexual conflicts. Sometimes women delay first intercourse in order to protect themselves against males that pursue only short-term sex and women developed a commitment scepticism bias. Males developed a sexual over-perception bias in order not to lose any single sexual opportunity. Both sexes have strategies to protect against sexual competition and infidelity (see for references Buss, 2008 p. 322–354). While these strategies often are successful in natural environments, we assume that in anonymous digital contexts deception of a possible mate – wherever in which aspect– is harder to detect given the absence of a common social field (no common friends, sometimes no face-to-face interactions). Digital “beauty-filters” are popular software applications in modern mobiles designed to increase the physical attractiveness in just a few instances. By using these applications, people try to deceive a possible mate about one’s own genetic fitness. Sometimes people even use images of other persons.

In detail we hypothesise that *female mating preferences* will shape the online mating market as follows: (i) a high level of education will be demanded more in men than it is in women; (ii) if there are men of different races, white men will be more eligible than males of colour; (iii) high-status men will look for younger women more than lower status men do; and finally; (iv) men will show in their personal ads more signs of physical strength than women do. *Men’s mating preferences* for numerous, young and physically attractive women will shape the online mating market as follows; (v) men will use dating platforms, hoping to extend the number of sexual partners, more than women do; and (vi) being of an older age is for women less favourable than for men.

The *interaction of male and female sexual strategies* will have the following consequences in the online dating market; (vii) women will receive more requests than men do, and (viii) will receive more responses to their own requests than men do; (ix) women will be more self-centred in their profiles and communication than men; (x) highly attractive women will give fewer responses to requests than less attractive women; (xi) compared to men, older women will use dating services more frequently than younger women do.

With respect to *increased sexual wellbeing*, we expect that; (xii) couples who meet in online settings will be more satisfied with their relationship than offline couples are (given the higher number of choices). With respect to *sexual conflicts and risks* to sexual wellbeing, we expect that; (xiii) women to be more frequently subjected to sexual deception because men are expected to display more emotional commitment than they actually feel (and a less possibilities for women to detect deception). We furthermore expect that; (xiv) using digital sexual dating services can be associated with or driven by psychological problems, such as feelings of loneliness or low self-esteem and finally; and (xv) that people who use online dating services show risky sexual behaviour more than others, e.g., unprotected sex.

To evaluate these hypotheses, we conducted a selective review of recent studies on digital sexual dating services.

## Methods

We first conducted an extensive literature research. We performed the literature research using the PubMed database. Given that online dating services constitute a rapidly changing market, we limited our literature research to studies with a publication year from 2015 to 2021. The search was restricted to publications written either in English or German. We used the following search terms: online dating (525 hits); infidelity, online (24 hits); sexual risk behaviour, online dating (51 hits); dating applications (1,058 hits); relationship, satisfaction, and online (910 hits). Our search string was as follows: Online Dating OR (Infidelity AND online) OR (sexual risk behaviour AND online Dating) OR Dating applications OR (Relationship AND satisfaction AND online). Every string was searched alone, there were 7 duplicates. In total, we found 2,568 publications in this initial step.

**FIGURE 1:**
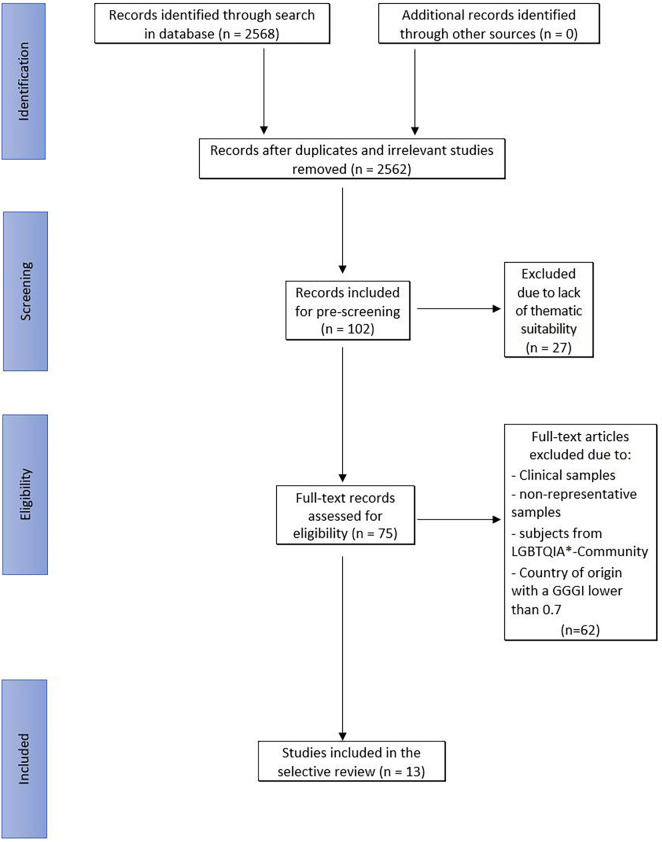
Flow diagram of the selective review according to Page et al. (2021).

Using several additional selection procedures and excluding criteria, we consecutively reduced the sample (Figure 1). First, studies with titles that did not match the topic of the current study were excluded. Duplicates were eliminated. We furthermore excluded studies with clinical samples, non-representative samples and samples with probands from the LGBTQIA* Community. Studies with clinical and non-representative samples had to be excluded to ensure a generalisation regarding the healthy population of the respective state as far as possible. Sexual selection theory provides no assumptions regarding sexual minorities, like members of the LGBTQIA* community. We therefore excluded studies with these samples. Furthermore, we excluded studies that were published in a country with a global gender gap index (GGGI) under 0.7 (Schwab et al., 2019) from this selective review. For this cut-off value we took the GGGI of Western Europe (0.767) and North America (0.729) into consideration (Schwab et al., 2019). Given the prior assumption that gender inequality restricts “free” dating behaviour (i.e., less driven by society’s constraints), we used this cut-off value to focus our review on samples from a more liberal dating culture. However, generalisability of our review is thereby limited to the more individualistic cultures of the Western world. On the other hand, this restriction increases comparability between studies included in the review.

Given that our review is focussed on sexual strategies in online dating, articles dealing with sexuality in the context of social media (Instagram, for example) were excluded. Although social media use in many cases leads to sexual interactions, social media are first designed for other purposes, making it more complicated to study sexual strategies in this area. Furthermore, articles that focussed on only one aspect of online dating (like alcohol abuse) were excluded as well. Furthermore, we were forced to exclude some articles because they were not available online. After applying these selection procedures, 13 articles remained. In a second step, we then reviewed the remaining 13 studies according to our study hypotheses.

## Results

All the studies included in the review were exclusively cross-sectional studies. In the following, we report the findings of the reviewed studies with respect to the hypotheses as stated in the introduction (see also Table 1).

**TABLE 1:** Studies included in the selective review.

Authors	Title	Study design	Sample size	Measurements	Main results
Harris and Aboujaoude (2016)	Online friendship, romance and sex: properties and associations of the online relationship initiation scale	Cross-sectional design	713 subjects	Online relationship initiations scale (ORIS)	Men report that they have failed to establish as many romances and sexual relationships as they had expected; people with problems in the offline social context benefit from online services
Lee et al. (2019)	Effects of self- and partner’s online disclosure on relationship intimacy and satisfaction	Cross-sectional design	189 subjects	Online self-disclosure scale, self-report measure and perceived relationship quality components; social media profiles	Greater online disclosure was associated with lower intimacy and relationship satisfaction. Offline disclosure is associated with the contrary.
Martins et al. (2016)	Infidelity in dating relationships: gender-specific correlates of face-to-face and online extradyadic involvement	Cross-sectional study	783 subjects	Questionnaire regarding social demographics and information about relationships, Extradyadic Behaviors Inventory (EBI), Attitudes toward infidelity scale und Investment model scale	Different factors such as previous infidelity, dissatisfaction in the current relationship, positive attitudes toward infidelity and high quality of alternatives (exclusively in women) are associated with emotional and sexual EDI online.
Navarro et al. (2020)	Psychological correlates of ghosting and breadcrumbing experiences: A preliminary study among adults	Cross-sectional study	626 subjects	Self-report questionnaire	People who experienced breadcrumbing or combined forms reported less life satisfaction, more helplessness & self-perceived loneliness. Regression model shows that experiencing breadcrumbing significantly increases the likelihood of experiencing less satisfaction in life and of feeling lonelier and more helpless. No significant relation was found between ghosting and any of the given psychological correlates.
Potarca (2021)	The demography of swiping right. An overview of couples who met through dating apps in Switzerland	Cross-sectional study	3245 subjects	Questionnaire	Online couples have a stronger need to move in together; women who met their partner online have a stronger desire to have a child in the next 3 years compared to women who met their partner offline; online couples who do not live together are significantly more satisfied than offline couples who do not live together; exogamy is more likely among dating app users in terms of education level; online couples travel significantly more distance to see each other.
Rochat et al. (2019)	The psychology of “swiping”: A cluster analysis of the mobile dating app Tinder	Cross-sectional design	1159 Tinder users	Problematic Tinder Use Scale (PTUS), Questionnaire assessing the pattern of Tinder Use, Short Happiness und Depression Scale (SDHS), Cybersex Motives Questionnaire (CMQ), Sexual Desire Inventory (SDI), Experiences in Close Relationships- Revised (ECR-R), Short UPPS-P Impulsivity Behavior Scale, Singe-Item Self-Esteem Scale (SISE)	Identified were the following four reliable clusters: Regulated, regulated with sexual desire (both low level of problematic use), unregulated-avoidants (intermediate problematic use), unregulated-highly motivated (high level of problematic use). Differences regarding gender, martial status, depressive mood and use patterns between the clusters.
Sedgewick et al. (2017)	Presenting your best self(ie): the influence of gender on vertical orientation of selfies on Tinder	Cross-sectional study	665 Tinder profile pictures	Objective rater estimated the vertical orientation of profile pictures, analysis via Welch’s ANOVA	Men are taking pictures more likely from below, women more likely from above.
Tsai et al. (2019)	Is online partner-seeking associated with increased risk of condomless sex and sexually transmitted infections among individuals who engange in heterosexual sex? A systematic narrative review	Systematic narrative review	25 Studies	Review using Google Scholar, PubMed, PsycInfo, Web of Science und Ovid Medline	58% of the studies show that online dating is associated with condomless sex or inconsistent condom use. 16% show that online dating is a protective factor. A 50% of the studies found no association between online dating and positive STI status, 36% found a positive association, 7% showed a negative association and 7% showed no clear results.
Whyte et al. (2018)	Do men and women know what they want? Sex differences in online dater’s educational preferences	Cross-sectional study	41.936 RSVP Accounts from which data were collected		Women were more likely to have specified education level as desirable in a potential partner; women show higher minimum standards for education compared to men.
Bruch and Newman (2019)	Structure of online dating markets in United States cities	Quantitative study	Dating website with more than 4 Mio. active users	Network analysis (Newman, 2018). Community detection (Fortunato, 2010)	Women respond to messages from men at a lower rate; women receive more responses from men when they contact men in older submarkets
Bruch and Newman (2018)	Aspirational pursuit of mates in online dating markets	Quantitative study	No details given	Estimation of “desirability” via communication patterns	Women are receiving more messages; desirability varies depending on age (stronger in women); education is more strongly associated with desirability in men; men responding to a women’s message is more likely than vice versa
Coor et al. (2019)	Sexually transmitted disease, human immunodeficiency virus and pregnancy testing behaviors among internet and mobile dating application users and nonusers	Cross-sectional study	Data from two market research datasets	Self-reports of the participants from the market research data sets	Higher rates of HIV and STD testing among users. Among 18–24 year olds, testing is significantly lower than among non-users; dating app users are more likely to have taken pregnancy tests in the last year.
Davis and Fingerman (2016)	Digital dating: online profile content of older and younger adults	Cross-sectional study	4000 online dating profiles	Quantitative content analysis of online dating profiles using LIWC software	Younger people are more likely to use 1st pers. singular pronouns and words from the categories: work, achievements, and negative emotion; men are more likely to use 1st pers. plural pronouns and words from the category work.

Findings related to *female mating preferences*:

*(i) A high level of education will be demanded more in men than it is in women.* Applying a detailed network analysis of messaging patterns and demographic variables in a data set of a huge dating platform, Bruch and Newman (2018) found that a high level of education is demanded more in men than it is in women.
*(ii) If there are men of different races, white men will be more eligible than males of colour.* In the same study (Bruch and Newman, 2018), it was found that white men are more eligible than black men.
*(iii) High-status men will look for younger women more than lower status men do.* In a subsequent analysis, the same authors (Bruch and Newman, 2019) found that high-status men look for younger women more than lower status men do.
*(iv) Men will show in their personal ads more signs of physical strength than women do*. One study analysed more than 900 self-portraits (“selfies”) that male and female Tinder users chose for their personal profiles (Sedgewick et al., 2017). Basically, they analysed whether males and females differ in the way they orient selfies to manipulate how they want to be perceived by the opposite sex. They found that males more often orient their selfies from below presumable to appear taller and more powerful than the viewer, whereas women were found to orient their selfies more often from an above perspective putative to appear shorter and to flatter the figure. Men manipulate the perspective of the viewer (from below) more often than women (from above), indicating that men guided their self-portrayal more according to female preferences than vice versa.

Findings related to *men’s mating preferences:*

*(v) Men will use dating platforms, hoping to extend the number of sexual partners, more than women do.* By means of an online survey with more than 700 participants, it was found that men use dating platforms, hoping to extend the number of sex partners, more than women do; however, they do not succeed in this as much as they expected to Harris and Aboujaoude (2016). Moreover, Martins et al. (2016) found that men more than women use online dating platforms for extradyadic sex.
*(vi) Being of an older age is for women less favourable than for men.* The above-cited network analysis of messaging patterns revealed in addition that being older is for women less favourable than for men (Bruch and Newman, 2018).

Findings related to *interaction of male and female sexual strategies:*

*(vii) Women will receive more requests than men do*. Two studies that analysed online communication patterns in huge samples reported that women receive more requests than men do (Harris and Aboujaoude, 2016; Bruch and Newman, 2018). In particular, Bruch and Newman (2018) found that more than 80% of first messages were sent from men.
*(viii) Women will receive more responses to their own requests than men do*. This was also reported by the study of Bruch and Newman (2018).
*(ix) Women will be more self-centred in their profiles and communication than men*. The study of Davis and Fingerman analysed 4,000 profiles of two popular websites by means of a linguistic inquiry and word count software. The authors found that women are more self-centred in their profiles than men are (Davis and Fingerman, 2016).
*(x) Highly attractive women will give fewer responses to requests than less attractive women do*. Bruch and Newman (2018) calculated desirability (so called page rank) of individual users in their huge sample and found that highly attractive women respond less often than less attractive women do.
*(xi) Compared to men, older women will use dating services more frequently than younger women do.* Bruch and Newman found in their subsequent analysis that in younger people, more men than women use online dating services; however, in older people this difference decreases (Bruch and Newman, 2019).

Findings related to *sexual wellbeing*:

*(xii) Couples who meet in online settings will be more satisfied with their relationship than offline couples are.* Contrary to our hypothesis Potarca (2021) found in a sample of more than 3,000 partnered individuals (based on an extensive inquiry among the general population of Switzerland) “no differences between couples initiated through dating apps and those initiated elsewhere regarding relationship and life satisfaction.”
*(xiii) Women are more frequently subjected to sexual deception than men are.* Similarly, we did not find evidence that women are more at risk of being sexually cheated than men are in digital contexts as revealed by an online survey with more than 600 individuals (Navarro et al., 2020).
*(xiv) Using digital sexual dating services can be associated with or driven by psychological problems, such as feelings of loneliness or low self-esteem.* Performing an online survey on more than 1,000 Tinder users, one study reported a “high level of problematic use” in nearly 1/3 of the sample. In these individuals, using sexual dating services is associated with psychological problems, such as feelings of loneliness or low self-esteem in some cases (Rochat et al., 2019).
*(xv) Users of online dating services show risky sexual behaviour more than others.* There is conflicting evidence concerning whether people who use online dating services show risky sexual behaviour more than others. Whereas Harris and Aboujaoude (2016) found a willingness to engage in infidelity or unprotected sex more in online dating users

(particularly in men) than in controls, Tsai et al. (2019) in their systematic review of 25 studies found no systematic association between condom use, sexually transmitted disease and engagement in online digital dating services.

## Discussion

This selective review evaluated a total of 13 cross-sectional studies on digital dating services. Evolutionary psychological reasoning and some hypotheses, which we drew from this, drove our analysis. Taken together, our selective review supports the notion that human sexuality is not going to change fundamentally on account of the rising popularity of digital dating services. The hypotheses that led our review covered four broader topics: (a) female mating preferences, (b) male mating preferences, (c) interactions between male and female mating preferences and finally, and (d) sexual wellbeing. The reviewed studies provided supporting evidence particular to hypotheses regarding mating preferences (a–c), which were derived from evolutionary psychological reasoning. Our hypotheses regarding sexual wellbeing received only partial support.

### Female Mating Preferences

Some of the reviewed studies provided findings that are in accord with evolutionary psychological reasoning. These studies report that women in the digital mating market appreciate men of higher education and of white colour. Both attributes are associated with higher socio-economic status in many countries. High-status men in turn look for younger women, as one study reported. This indicates that high-status men in digital mating markets are aware of female mating preferences. Both findings (preference for high-status males and pursuit of younger women by high-status men) have previously been found in non-digital mating markets (Buss, 1989b; Grammer, 1992; Buss and Shackelford, 2008; Iredale et al., 2008; Vohs et al., 2014; Ponseti et al., 2018). One of the reviewed studies concluded that men try to look taller and more powerful as they orient their selfies more often from below (Sedgewick et al., 2017). In fact, women prefer males who are physically more powerful and taller (particularly in a short-term mating context). Again, this has been found already in the non-digital mating market before (Frederick and Haselton, 2007). Obviously, men act according to female preferences for physical dominance and display as much as possible of this trait. This in turn is sexually rewarded: physically powerful men report more sexual partners than less powerful men do (Frederick and Haselton, 2007).

We assume that female mating preferences (like male mating preferences as well) are shaped by sexual selection and modulated by culture and actual conditions of the mating marked (in terms of demand and supply). The findings discussed so far indicate that humans act according to female mating preferences in (sometimes anonymous) digital dating arenas more or less similar to real-world encounters.

### Male Mating Preferences

According to sexual selection theory, males have more fitness benefits from having numerous sexual partners than females do. Therefore, males are predicted to pursue more sexual partners than females. In humans, this is particularly evident when looking at gender differences regarding interest in short-term sex (Clark and Hatfield, 1989; Voracek et al., 2005; Gueguen, 2011). This has been found outside the digital market area previously and appears to be true in the digital dating market in the same manner (Harris and Aboujaoude, 2016; Martins et al., 2016). Male fitness benefits from high numbers of sex partners and from having young sex partners given that the reproductive capacity of a young female is higher than that of an older female. Therefore, men appreciate youthfulness in their female partners much more than vice versa (Buss, 2008, S. 114). Again, what has been found in real-word mating with respect to male mating preferences is mirrored in the digital mating market (Bruch and Newman, 2018).

### Interaction of Male and Female Sexual Strategies

A striking gender difference was reported by the study of Bruch and Newman in that 80% of first messages were sent by men (Bruch and Newman, 2018). Given that in the study of Bruch and Newman the numbers of male and female participants were roughly similar, the reported difference cannot be due to a limited female supply. It rather suggests that males are much more active, if not impatient, in establishing contacts. This male over-activity might be the result of both, (i) the possibility to anonymously interact with several women at the same time (driven by the strategy to find as many mates as possible and the lack of social control) and (ii) the lack of concealed signals from women that help men to focus on those mates with prospect of success. Male over-activity in turn puts females in a more comfortable position, allowing them to define the rules of the game more according to their own needs. One possible consequence of this is that women are more self-centred in their profiles and communication (Davis and Fingerman, 2016). A pattern that is pronounced in high attractive women. These women respond even less to male requests than less attractive women (Bruch and Newman, 2018). These findings are in accord with sexual selection theory predicting the higher investing sex to be choosier and the lower investing sex to be more competitive in its efforts to sexually access the higher investing sex. We predicted the respective findings for the digital mating arena because similar observations have been made in real-world scenarios previously – and, of course, because of our overall hypothesis that important variances in human mating strategies have been shaped in ancient times. However, interactions between male and female mating strategies are complicated and require a closer look, even though the data of our selective review on this was scarce. In real-word scenarios, the supply of males is an importance factor that modulates the female inclination to engage in short-term sex. If there are fewer males than females in a given mating market, females tend to be more willing to engage in short-term sex; “sex becomes cheap” (Barber, 2000; Schmitt, 2005; Xing et al., 2016). Conversely, “sex becomes expensive” when there is more male demand. Possibly, the observed self-centredness of women in digital dating markets is caused by the dynamic between supply and demand. As noted above, a surplus of male demand can be experienced in a mating market even if absolute numbers of males and females are equal simply because one sex is more impatient in its efforts to establish contacts.

However, it is not self-evident that a surplus of male demand in the digital (as well as in the real-world) market is only driven by male’s (ancient) strategy to find as quickly as possible as many mates as possible. In a seminal review, Baumeister and Twenge (2002) showed convincing evidence that women work together to restrict male’s sexual access to females (in order to get as much as possible in exchange for sex). One strategy is to hide, respectively, to obscure a female’s own sexual interest. Women are influenced by other women (mothers, sisters, girlfriends, etc.) which makes them feel uncomfortable when openly showing their own sexual needs. This cultural force, in addition to adaptations shaped by sexual selection and the specific conditions of anonymous digital dating, might be one further reason why 80% of first messages were sent by men.

### Effects of Digital Dating Services on Sexual Wellbeing

Contrary to our expectations, we found no studies that reported high numbers of persons being victims of sexual deception (as described above). However, we found no study that investigated this topic from an evolutionary psychology viewpoint directly. Taken together, the reviewed studies provided mixed information about whether using digital dating services might lead to increased sexual wellbeing or not. In Tinder users, feelings of loneliness or low self-esteem were found quite often (Rochat et al., 2019); others reported a willingness to engage in infidelity or unprotected sex, particularly in male users of some other data bases (Harris and Aboujaoude, 2016); however, Tsai et al. (2019) found no evidence for this in their systematic review. Moreover, a systematic comparison of couples who have met online vs. offline based on a large representative sample reported no difference regarding the quality of the relationship. That is, differences concerning sexual wellbeing between the online and offline dating world might not be as big as they were sometimes assumed, maybe with the exception that some individuals with specific problems might be attracted by particular dating services. We propose that the specific interactions between personality characteristics and characteristics of certain dating services that may lead to problems of sexual wellbeing should be investigated in future research. It is possible that the benefits of digital dating services are underestimated as well. It was found that online couples are not better off than offline couples. However, it is possible that many people are in a stable relationship or experience sexual intimacy thanks to the use of digital dating.

### General Limitations

The findings of this selective review are limited by the fact that the studies included in our review were not designed to test evolutionary psychological hypotheses. This has led to a type of methodological cherry-picking in the sense that we just looked at the reviewed studies for evidence that seemed to match with (or contradict) our expectations. One problem with this approach is that the samples of the reviewed studies were of quite different origins. Some studies were based on representative samples of the general population, whereas others focussed on particular individuals, e.g., Tinder users or individuals in committed relationships. Moreover, our review covers different types of dating services; some of them offer opportunities for short-term dating, whereas others focus on long-term dating. These aspects also influence the operational sex ratio. There is ample evidence in evolutionary psychology that people experience different sexual preferences and apply different strategies depending on whether they are looking for a short-term mate or a long-term mate and depending on whether there is a surplus of males or females in a given mating arena. This leads to some limitation in the reported findings given that our hypotheses were found to be proved sometimes in one sample type but not in another sample, and vice versa. In most cases this was influenced by the fact that not all studies we reviewed provided information regarding all our hypotheses.

### Closing Remarks and Recommendation for Future Research

Digital sexual services go far beyond pure dating platforms: cybersex via webcam, chatting with sex bots, sex robots and self-broadcasted sexual content are recent developments in a fast-developing digital market. In the light of the findings reviewed here so far, we assume that these developments and future digital sex services will be driven by both, new technologies and ancient sexual strategies of men and women; however, it is hard to predict how both factors will interact in future. It should be subject of an ongoing research. To circumvent the limitations of the present selective review we recommend that future studies should directly test hypotheses that are derived from evolutionary psychology in samples that engage in different types of digital sexual services.

## Data Availability Statement

The original contributions presented in the study are included in the article/supplementary material, further inquiries can be directed to the corresponding author.

## Author Contributions

JP, KD, and AS contributed to conception and design of the study and wrote sections of the manuscript. KD and JP organised the database. JP wrote the first draft of the manuscript. All authors contributed to manuscript revision, read, and approved the submitted version.

## Conflict of Interest

The authors declare that the research was conducted in the absence of any commercial or financial relationships that could be construed as a potential conflict of interest.

## Publisher’s Note

All claims expressed in this article are solely those of the authors and do not necessarily represent those of their affiliated organizations, or those of the publisher, the editors and the reviewers. Any product that may be evaluated in this article, or claim that may be made by its manufacturer, is not guaranteed or endorsed by the publisher.
